# Supplementary material for: Control of filament length by a depolymerizing gradient
Source: PLoS Comput Biol. 2020 Dec 4;16(12):e1008440. doi: 10.1371/journal.pcbi.1008440 (PMC7744062; doi:10.1371/journal.pcbi.1008440)
Supplement: Attachment — Submitted filename: reviewer_response v2.docx [file pcbi.1008440.s001.docx]

Response to reviewers

The reviewers have made a number of interesting and useful comments that have helped improve the overall quality of the manuscript and we are grateful to them for that. Below we give the reviewer comments (in gray) and our responses (in black) to them. We also describe the relevant changes to the manuscript, which were made in response to the specific comments.

Reviewer #1: In this article, the authors aim to establish a stochastic model of flagella growth and recession by introducing two separate but related methods of depolymerization that are dependent on the instantaneous length of the flagella. In these models, tubulin is transported to the free end of the flagella at a constant rate while a depolymerizing protein establishes a nonuniform concentration profile within the flagella that increases with distance from the base. The authors consider two types of concentration profiles: linear and exponential. In both cases, the probability distribution of flagella
lengths is calculated as a function of the profile lengths scales and a defined dimensionless parameter. The statistical results, namely steady state length and relative noise, are compared to experimentally measured values. While steady state length is found to be in good agreement, calculations of relative noise are a full two orders of magnitude below the experimental values.

I find the foundations of this article to be strong and well-motivated. Polymer dynamics is a rich and complex field, and the ubiquity of polymers throughout biology makes understanding how these dynamics function and are possibly regulated relevant for the study of most cells. The study performed here is narrowed to focus on the assembly and disassembly of flagella and other filamentous structures. The method used is a simple chemical master equation that expresses the length of such filaments as a stochastically evolving quantity. The resultant math is presented in a compact and easy to comprehend manner that highlights the major results of the study well. Importantly, the authors also present possible experiments that could be performed to test some of the predictions made by their models.

Despite these qualities, there are several areas of the article that should be addressed. In particular, I find two distinct major issues I feel need to be examined in some way within the article itself as well as a few other smaller remarks that I believe would make the paper stronger with minimal additional effort.

Major issues:

1) There are several different timescales inherent within the system the authors are attempting to model. While the authors note their approximation that the binding of depolymerizing protein to the tip of the filament is the rate limiting step when compared to the time needed to diffusively find the filament tip and the time to remove the terminal subunit, there are many more timescales that remain unmentioned. In the case of the linear gradient, there is the rate at which new depolymerizing protein attaches to motors at the proximal end, the time required for a motor to traverse the length of the filament, and the time required for a protein to diffuse back to the proximal end from the tip. The exponential gradient case also adds the rate at which diffusing protein in the filament rebind to already moving motors. These timescales need to be either included in the model or shown through experimentally measured values to be negligibly short. As an example, if the time to transport depolymerizing protein from the proximal to terminal end of the filament is of the same order as or longer than the time scale of the dynamics of the filament itself, then the concentration of depolymerizing protein at the tip will be a function not of the length of filament but of the length of the filament at a time in the past. This delayed reaction could have significant consequences on the steady state distribution of filament lengths. As such, the authors need to go back through their model, identify all possibly relevant time scales involved, and either include their effects in the model itself or show that they are realistically negligible.

The reviewer is correct to point out that there are a number of different processes at play and a separation of timescales needs to be established. Indeed, our model assumes that the timescale associated with the relaxation of the gradient, i.e., the time to reestablish the gradient after a change in filament length has occurred, is fast compared to the timescale associated with filament growth. Wether or not this separation of time occurs depends on the values of all the different rates brought up by the reviewer. Instead of trying to make estimates of these rates as most are unknown in the cellular milleau, we take our cue from eperiments on flagellar transport in Giardia and Chlamydomonas cells.

Separation of time scales associated with flagellar growth and depolymerizer gradient dynamics was recently established in Giardia cells by MacInally et al. [figure 8 in ref. 19]. In Giardia, the growth rate of flagella is about one micron per hour. The microtubules in the flagella are depolymerized by kinesin-13, which is shown to exhibit an exponential gradient along the length of the flagellum. When fluorescently labeled kinesin-13 is photobleached in the flagellum (in the region 1 micron away from the tip), it is found that the timescale to recover the gradient is about one min, which is much faster than the rate of flagellar growth. Similar data was found for the kinesin-2 gradient in Chlamydomonas, which is linear along the flagellum [figure 5 in ref. 25]. The rate of flagellar growth in these cells is about 1 micron/min. On the other hand, the gradient recovery time after photobleaching is found to be about 3 sec. While kinesin-2 is not a depolymerizing protein, in the absence of measurements of a depolymerizing protein gradient in Chlamydomonas, we take these numbers to be representative of a protein gradient formed by the combined effect of diffusion and directed transport by motors.

Since available experimental data point to a separation of timescales between filament dynamics (slow) and depolymerizing gradient dynamics (fast), we treat the gradient dynamics using an adiabatic approximation, with the gradient simply being a function of the instantaneous filament length. We have added a paragraph in the manuscript that describes the experimental evidence for the separation of timescales, and clarified the nature of the approximation that this implies.

2) Closely related to the issue of time scales is the issue of noise sources. In the chemical master equations presented in Eqs. 7 and 13, the only stochastic parameter is the filament length. However, concentration profiles of chemicals such as proteins are famously stochastic themselves. Fluctuations in the binding of filament subunits or depolymerizing protein to the motors can cause similar fluctuations in the assembly and disassembly rates of the filament respectively. Additionally, the depolymerizing proteins move through the filament diffusively once they detach from the motors. Diffusive fluctuations in the concentration can not only affect the concentration at the tip but also the rebinding rate to motors throughout the filament in the case of the exponential profile. As such, the assembly rate and depolymerizing protein concentration should themselves be treated as stochastic unless their inherent fluctuations can be shown to be either negligibly small or occur over timescales fast enough that they can be well approximated by their mean values. The addition of these noise sources could help explain the 100-fold discrepancy between the predicted and experimentally measured noise, though such a drastic shift in the predicted noise would itself be a concern unless thoroughly explored and understood.

Indeed, the depolymerizer gradient itself is stochastic in nature. But, as discussed above, the relaxation timescale of the gradient is faster than the timescale of assembling the filament. So, we expect the fluctuations to be fast enough that we can consider the gradient to be well approximated by the mean value.

For the fluctuations in the assembly rate the relevant timescale is the time between two IFT trains, which are measured to be of order seconds in Chlamydomonas and in Giardia [figure 4 in ref. 19]. Given the measured growth rates of a micron per minute in Chalmydomonas and a micron per hour in Giardia, this implies a few hundred tubulin dimers arriving with each train. In other words, the timescales at which individual building blocks (tubulin dimers) arrive at the tip, where they are incorporated into the growing filament, are small compared to the timescales of length fluctuations. This justifies using a constant rate of assembly. We have added clarifying sentences to this effect in the manuscript.

Therefore, while our variance from the model does not agree with experimental results, we do not believe that these fluctuations are the cause of these deviations. It should be noted that there are several other sources of noise presented in the experiments. Most notably, the results were obtained with light microscopy and the diffraction limit of the microscopes would produce errors in measuring length of the order seen in the data. We feel that more careful experiments are necessary before we can be certain about the size and the nature of the length fluctuations are. Furthermore, we hope that our article can point out the importance of measuring these fluctuations carefully, which has not been done to date (to our knowledge).

Minor issues:

1) In the introduction the authors discuss experiments in which a flagellum of a Chlamydomonas cell is cut and the other shrinks in response until both reach the same length and regrow in unison. This is presented as a compelling argument for the validity of the shared monomer pool model, yet it is unclear as to whether the model presented here can reproduce this phenomenon. If it can, a visualization of such an occurrence using the model would strengthen the argument for the model’s overall validity. If not, this shortcoming should be addressed in the discussion.

The analysis suggested by the referee was published in Fai et al [figure 4 in ref. 18] where the predictions of the rate equations that describe the dynamics of the mean length of the two flagella are compared to the regeneration experiments, and good agreement was found.

2) In Eqs. 8 and 9, 1/Z is used as a normalized factor, but in Eqs. 14, 15, and 17 there is the factor A that is not explicitly stated to be a normalized factor, though it seems fairly obvious from context that that is its purpose. These notations should be uniform across the article with all equations using either 1/Z or A as the normalization factor.

We thank the reviewer for noticing the typo. We have corrected it in the manuscript.

3) When discussing Fig. 3E and 3F, the author note “As the fraction of monomers in the filaments decreases the difference between the predictions of the finite and large monomer pool case also decrease, as expected.” This should be shown in Fig. 3E and 3F by including multiple lines for finite monomer pools with varying fractions of the monomers being held within the filament.

The reviewer raises an interesting point that requires some discussion. There are two parameters in the model, the length scale of the gradient lambda, and the dimensionless parameter *QN*, and the fraction of monomers in the filament can be made small, by making one or the other parameter small. So, our figure shows the agreement between a finite pool and an infinite pool model getting better and better as lambda gets smaller, i.e., as the fraction of monomers in the filaments becomes small. To make this point more clearly, we have now added two inset figures which show how decreasing QN and therefore the fraction of monomers in filaments, while keeping lambda fixed, also leads to better and better agreement between the finite and infinite monomer pool results. Hopefully this makes more clear the point about the agreement between the finite and infinite pool models we wish to make.

4) In line 313 the authors state “which we find to be an excellent approximation (difference) of the mean.” I am unclear as to why the word “difference” is included in parentheses here as it seems to serve no purpose in the sentence.

We thank the reviewer for pointing out the typo. We have fixed it in the manuscript.

Reviewer #2: In this paper the authors address the question how cells can control the length of multiple filamentous structures. In order for the structures to maintain a precise length, length-dependent assembly and/or disassembly rates are required. In their manuscript, the authors focus on length-dependent disassembly rates mediated by a depolymerase which is transported along the filamentous structure. Specifically, they assume that the depolymerase reduces the size of the filament with a typical rate once it reached the tip of a filament. Depending on the underlying dynamics of the depolymerase, distinct concentration profiles (of the depolymerase) emerge along the filament and thereby also different tip-occupations, which give rise to different effective disassembly rates. The authors model the depolymerase in an ad-hoc manner by considering different (typical) concentration profiles, namely a linear and an exponential concentration profile. They calculate the structure size distribution by solving the master equation for the underlying rate equation in the case of finite and infinite resources. They compare their theoretical results to experimental findings for biologically reasonable parameter estimates, and find good agreement for the mean structure size but the variance seems to significantly differ. A key prediction of their model is that the mean stationary state length of filaments in the linear gradient model depends linearly on the total amount of available assembly structures, whereas in the exponential gradient model the mean steady stationary length has a logarithmic dependence on the amount of available assembly structures.

Major Criticism

Since the authors derive the probability distribution of the underlying rate equations, their result for the variance is an important result of their analysis. However, they only briefly discuss the possible reasons for the clear deviations between the model and the experiments. From the perspective of the model, a natural explanation seems to us that the dynamics of the depolymerase is considered ad hoc via the concentration profiles. Actually, however, the depolymerase itself performs a stochastic movement. We would like to ask the authors to complement their discussion with stochastic simulation that explicitly considers the stochastic dynamics of the depolymerase.

The reviewer is correct in pointing out that the depolymerase molecules are also subject to stochastic dynamics. However, the reason we don’t consider these dynamics is because of the separation of timescale between the relaxation of the gradient, i.e., the time to reestablish the gradient after a change in filament length has occurred, and the timescale associated with adding or removing a filament subunit. As discussed in detail in the response to the similar comment made by Referee 1, experiments on flagella in Giardia and Chlamydomonas cells find, via photobleaching experiments, that the relaxation dynamics of the protein gradients, formed by the combined effect of diffusion and transport, happen on time scales of seconds. The relevant time scales for flagellar growth are much longer, minutes (Chlamy) and hours (Giardia). So we use this observation to justify our adiabatic approximation in which the gradient dynamics is ignored. We therefore believe that the low noise in length fluctuations is a testable feature of this model, and we hope that this will encourage experimental groups to measure the length fluctuations more carefully. Current measurements using light microscopy are diffraction limited so the experimental errors are expected to be of the order of 100s of nanometers, which is comparable to published data.

The line of argument following line 146 is unclear to us. The authors use MCAK as an example for a linear relation between tip concentration, c(L), and effective depolymerization rate, ɣ(L). However, we would not consider MCAK to be the best example, because it actually performs a diffusive motion on the filament, which implies a flat concentration profile that does not correspond to the model assumptions (linear/exponential concentration profile) of the authors.

Indeed, MCAK performs diffusive motion on the filament and we did not mean to suggest that it is transported by motors, since no such data exists, to our knowledge. In the paper we only used MCAK experimental data to illustrate the point that the depolymerization rate of tubulin in the presence of depolymerizing molecule (such as MCAK), is expected to be proportional to the depolymerizer concentration at the tip c(L). We did not mean to imply that MCAK is the depolymerizing protein in Giardia or in Chlamydomonas cells, which served as the motivation for our theory. In fact, in Giardia it has been shown that the depolymerizer is kinesin-13 that is brought to the tip by intraflagellar transport, while in Chlamydomonas, no such depolymerizer has been identified (there are observation of kinesin-13 localization at the flagellar tip consistent with such transport, but only in cases when rapid depolymerization of the flagella occurred). We have changed the text to remove this point of confusion, as we certainly do not want to imply that MCAK is the depolymerizing protein involved in the length regulation of flagella.

The directed transport of molecular motors with on/off kinetics is often modelled with the totally asymmetric simple exclusion process with Langmuir Kinetics. In the limit of low densities, such an analysis yields to a linear concentration profile as considered by the authors. However, the effective depolymerization rate would be determined by a flux balance equation at the tip, ε c(L)[1-c(l)]=γ(L), which implies a nonlinear dependence on the tip occupation. We would like to authors to comment on the influence of nonlinearity in the depolymerization rate on their results.

Indeed, TASEP has been used to model motor dynamics, and as remarked by the reviewer, experiments on intraflagellar transport suggest that the low-density limit of TASEP is appropriate in this case. The depolymerizer rate suggested by the reviewer is correct assuming that the depolymerizing molecules brought to the tip of the flagellum by motors directly bind to the tubulin monomers before knocking them off the microtubule (as is the case of kinesin-8 [ref. 22]). However, in our model the depolymerizer molecules that bind to the tubulin at the tip, thereby inducing depolymerization, are not on the filament, but are molecules in the cytoplasm near the filament tip (our c(L) therefore does not refer to the concentration of these molecules on the filament, but in the cytoplasm in the vicinity of the tip of the flagellum). This scenario is supported by experiments in Giardia, which demonstrated the role of depolymerizing kinesin-13 in controlling length [ref. 19]. Kinesin-13 is not a motile kinesin and photobleaching experiments suggest that it is transported to the tip by intraflagellar transport, where we assume (as observed in Chlamy cells [ref. 22]) that the IFT trains fall apart releasing their cargo, of which kinesin-13 is one component. We have clarified this point of confusion in the manuscript to make sure it is clear that the mechanism of depolymerization is not that of the motile kinesin-8, but by a depolymerizer carried as cargo by IFT.

Minor Criticism

In line 269 they state that the steady state probability distribution must satisfy the relations given in line 271. We would suggest to add that the conditions given in line 271 correspond to the detailed balance condition which has to be fulfilled since the assembly process is time reversible. For a general stochastic process, we would not agree with the statement that the steady state probability distribution must fulfill the detailed-balance condition.

We thank the reviewer for pointing out this omission. We have made the necessary changes in the manuscript.

In Figure 3 E,F the biologically meaningful parameter range should be given so that one can judge whether the deviation between the finite and infinite pool model occurs for meaningful parameters.

We thank the reviewer for pointing out the omission. For Chlamydomonas, we know that the filament length is around 10 microns, so $\lambda$ corresponding to that length is the relevant parameter for both the models. For that $\lambda$, we can see that the linear gradient model differs about 30% between the finite and infinite pool, while the difference is about 5% for the exponential model. We have added this comment to the discussion of this figure.
